# MUCHA: multiple chemical alignment algorithm to identify building block substructures of orphan secondary metabolites

**DOI:** 10.1186/1471-2105-12-S14-S1

**Published:** 2011-12-14

**Authors:** Masaaki Kotera, Toshiaki Tokimatsu, Minoru Kanehisa, Susumu Goto

**Affiliations:** 1 Bioinformatics Center, Institute for Chemical Research, Kyoto University, Uji, Kyoto 611-0011, Japan; 2 Human Genome Center, Institute of Medical Science, University of Tokyo, 4-6-1 Shirokanedai, Minato-ku, Tokyo 108-8639, Japan

## Abstract

**Background:**

In contrast to the increasing number of the successful genome projects, there still remain many orphan metabolites for which their synthesis processes are unknown. Metabolites, including these orphan metabolites, can be classified into groups that share the same core substructures, originated from the same biosynthetic pathways. It is known that many metabolites are synthesized by adding up building blocks to existing metabolites. Therefore, it is proposed that, for any given group of metabolites, finding the core substructure and the branched substructures can help predict their biosynthetic pathway. There already have been many reports on the multiple graph alignment techniques to find the conserved chemical substructures in relatively small molecules. However, they are optimized for ligand binding and are not suitable for metabolomic studies.

**Results:**

We developed an efficient multiple graph alignment method named as MUCHA (Multiple Chemical Alignment), specialized for finding metabolic building blocks. This method showed the strength in finding metabolic building blocks with preserving the relative positions among the substructures, which is not achieved by simply applying the frequent graph mining techniques. Compared with the combined pairwise alignments, this proposed MUCHA method generally reduced computational costs with improving the quality of the alignment.

**Conclusions:**

MUCHA successfully find building blocks of secondary metabolites, and has a potential to complement to other existing methods to reconstruct metabolic networks using reaction patterns.

## Background

Living organisms in nature use a variety of substances that express both conserved and variable functions to survive. For example, genes and proteins have conserved sequences or motifs that usually express their essential functions, and some variable regions are known to provide varieties to the functions including immunity. Similarly, a variety of relatively small metabolites can be grouped into those common to many different species (primary metabolites) and those observed in a limited set of species (secondary metabolites). Secondary metabolites have been shown to be of great value in the classification and differentiation of fungal species [1]. For another example, plants produce over 200,000 secondary metabolites [2], some of which are known to function as toxins defending the organisms against pathogens, parasites and predators [3]. The physiological roles of many secondary metabolites are still unknown; however, some of them are important sources of drugs and industrial materials.

Many secondary metabolites are not yet known how they are synthesized or degraded, which can be referred to as “orphan metabolites” in metabolomic studies [4] by analogy with orphan genes in genomic studies [5]. These orphan metabolites can be divided into groups that share the same core substructure, originated from the same biosynthetic pathways. It is also known that many metabolites are synthesized by adding up the building blocks onto the other existing metabolites. Therefore, finding common and branch substructures from a group of compounds may narrow down the search space to identify their biosynthetic pathway compared with the prediction of pathway for each metabolite individually. This is our motivation of developing novel multiple chemical alignment (atom-atom mapping) algorithm, which is different from the ones for motif finding.

Multiple sequence alignment algorithms are valuable in finding conserved and variable patterns across a family of nucleic or amino acid sequences, and have been shown of major importance in bioinformatics. The concept of multiple alignment can also be applied into graph structures. Graph is a general data structure where some pairs of the objects are connected by links, and has been used for modeling biological networks [6-9], three dimensional structure of proteins [10-12], as well as molecular structures [13-21]. There have already been a number of multiple graph alignment or frequent subgraph mining methods [22-24]. Those techniques have their own strengths and weaknesses because of the variety of different requirements of finding substructures. Therefore different strategies have to be designed depending on the purpose of finding common substructures. For example, the common substructure of a set of polypeptides is obviously a peptide backbone. In most cases, however, “a peptide backbone” is not the proper answer for the researchers using the multiple alignment. The purpose of the multiple alignment is usually finding the conserved sequence of amino acid residues, which is why multiple “sequence” alignment method is suitable rather than multiple “graph” alignment.

On the other hand, some multiple graph alignment methods were developed for finding functional groups or substructures in chemical compounds responsible for ligand binding. Graph-based methods have strengths in identifying conserved substructures and generating atom-atom alignments. Many researchers proposed graph-based algorithms for obtaining the maximum common subgraph (MCS) [25] using clique-finding [26] and backtracking [27,28] techniques. The MCS problem is known as NP-hard, so most algorithms are not universally applicable. Therefore, the graph-based algorithms use some heuristics to effectively reduce the computational amount, to provide a specialized solution for the concrete properties of their problems arisen from special requirements. Recently, an evolutional algorithm-based approach has been proposed to solve multiple graph alignment [12], although it was optimized for three-dimensional protein structures and it still required many computational time even for relatively small molecules. More importantly, these multiple alignment methods perform 3D alignment (i.e., superposition) of molecules to deduce pharmacophores or the sites of molecular recognition, which should match different chemical groups with “similar” properties responsible for the binding to proteins that are usually oriented similarly among the group of ligands. In this context, “similar” chemical groups do not mean the chemical groups that can be converted to each other by enzyme reactions, but they typically mean electrostatic properties such as polar positive, polar negative, or hydrophobic. This strategy is apparently not suitable for finding metabolic building blocks.

**Figure 1:**
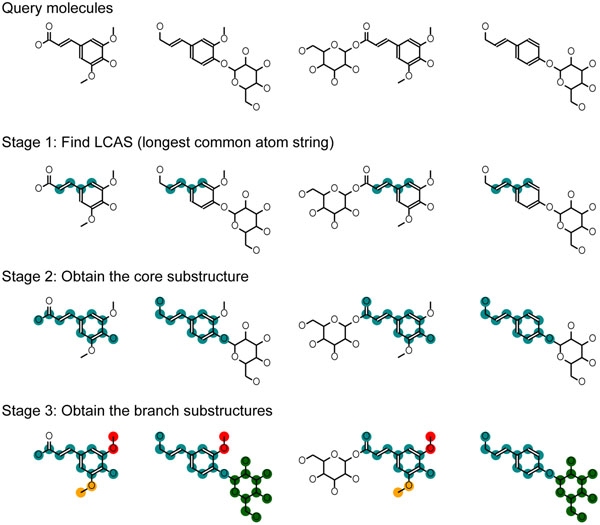
Overall procedure for the multiple chemical alignment

We propose in this study that the techniques for predicting metabolic origins require different strategy than that for motif finding or ligand binding. We thus developed the multiple chemical alignment (MUCHA) algorithm for assisting the metabolic pathway prediction. Our method was shown to be efficiently quick to apply for finding the core and branch substructures from large number of compounds. The main procedures in the MUCHA algorithm are: (1) obtain the longest common string of atoms, (2) extend the string to obtain the core substructure, (3) apply the similar strategy to the peripheral atom strings (Figure 1). We propose this method as the powerful tool to classify metabolites based on the building blocks and to facilitate the prediction of their biosynthesis pathways.

## Materials

**Figure 2:**
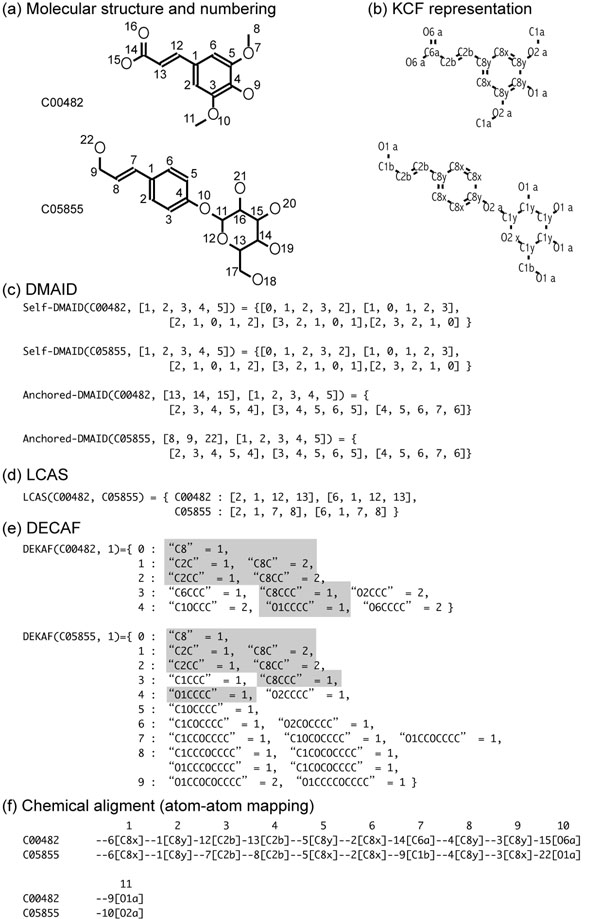
Examples of DMAID, DECAF, LCAS and the chemical alignment

Chemical structures of metabolites were obtained from the KEGG LIGAND database [29] (Figure 2a). C00482 and C05855 are the KEGG Compound IDs for the example molecules, sinapic acid and p-coumaryl alcohol 4-*O*-glucoside, respectively. KEGG Chemical Function (KCF) format represents chemical compounds as graphs G(V, E), where V and E are the sets of vertices and edges, i.e., the sets of atoms and bonds found in each molecule, respectively. The vertices (atoms) do not only contain atomic species information but are labeled by the KEGG Atom types [20], which describe the detailed information of atomic properties such as functional groups (Figure 2b). KEGG atom label consists of three letters, such as “C1a” meaning a methyl carbon. The first and second letters represent atom species and orbital environments, respectively. The third letter describes the surroundings of a given atom in terms of its bonded neighbors. The list of the KEGG atom typing is given in the Supporting Information. In this study, we refer to the full KEGG atom types consisting of the three letters as the “KEGG atoms”, up to the first two letters as the “atom classes”, and the first letter as the “atom species”. Note that hydrogen atoms are not usually described as the vertices, unless it is necessary to represent the stereochemistry, but the involvement of the hydrogen atoms is implicitly represented in the KEGG atoms. Also, the numberings of the atoms in the molecules we used, as described in Figure 2a, were not base on the IUPAC rules but were automatically assigned by the chemical structure drawing tools including ChemDraw and KegDraw.

## Methods

In this section, we first introduce the naïve extension of SIMCOMP [20], the effective pairwise alignment method, for the comparison. Consequently, we defined the two key methods for the proposed MUCHA method in this study, DMAID (distance matrix-based atom identifier) and DECAF (distance-embedded common atom fingerprint). Using these two, the MUCHA calculates the multiple chemical alignment through the process as described in Figure 1: [Stage 1] to obtain the longest common atom strings (LCAS), [Stage 2] to extend LCAS to obtain the core alignment, and [Stage 3] to conduct the similar strategy to the branch structures.

### MULCOMP as the naïve multiple chemical alignment tool

We designed the naïve version of the multiple chemical alignment method by assembling the SIMCOMP pairwise chemical alignments for all combinations of the molecules given as a query. We refer to this naïve method as MULCOMP. Since a SIMCOMP pairwise alignment is calculated independently from other alignments, there occurred many cases where the atom-atom mappings in some alignments conflicted to each other. In such cases, we removed the conflicting atom-atom mappings. This removing process is also applied to the branch alignment in MUCHA, and was thus explained in the later section in this paper.

### Distance matrix-based atom identifier (DMAID)

The first technique of MUCHA we applied was named as the distance matrix-based atom identifier (DMAID), which is used for characterizing the atom's or atom string's relative position. In this context, “distance” means the shortest path length between two atoms in a molecular graph. Distance 0 refers to the current atom. Distance 1 refers to the atoms directly bonded to the current atom. Distance 2 refers to the atoms bonded to the distance 1 atoms, and so on.

Example DMAIDs are shown in Figure 2c. We defined the two types of DMAIDs: self-DMAID and anchored-DMAID. Self-DMAID basically has the same structure as the distance matrix. The differences lie on that the self-DMAID only considers the atoms included in the given set of atoms (*i.e*., LCAS) in a molecule, and that self-DMAID is used for the distinction of the atom strings to obtain LCAS. Self-DMAID always becomes a square matrix. Take the atom string "1, 2, 3, 4, 5" of the molecule C00482 for example, the shortest path lengths from the atom 1 to the atoms 1, 2, 3, 4, 5 were 0, 1, 2, 3, 2, respectively. This way, shortest path lengths from the atoms in the atom string to their own were put in an array, which made a square matrix enabling the quick check of the topological identity among the atom strings. For instance, as described in Figure 2c, self-DMAID for the atom string "1, 2, 3, 4, 5" of the molecule C00482 was the same as the one for "1, 2, 3, 4, 5" of C05855. However, these two atom strings were not regarded as LCAS, because they were not the same in terms of the KEGG atom strings (“C8y-C8x-C8y-C8y-C8y” and “C8y-C8x-C8x-C8y-C8x”, respectively). If all the query molecules have the atom strings that are the same in terms both of the self-DMAIDs and the KEGG atom strings, then the atom strings were regarded as the common atom strings to obtain the core substructure.

On the other hand, the anchored-DMAID for an atom or atom string needs the other atom or atom string as the anchoring point(s), and were used to obtain the branch substructures using the LCAS as the anchor. An anchored-DMAID is a distance matrix of an atom string against another, therefore it is not necessarily a square matrix. For example, the anchored-DMAID for the atom string "13, 14, 15" of the molecule C00482 against the anchor string "1, 2, 3, 4, 5" is shown in Figure 2c. The calculation process is the same as that of the self-DMAID. Figure 2c shows another anchored-DMAID that was the same as the first one. After the core substructure was obtained, the anchored-DMAIDs were calculated for the short common atom string (SCAS) against the core. Different from the core substructures, if the atom strings are the same in terms both of the anchored-DMAIDs and the atom species strings, then they were regarded as the common strings to obtain the branch substructures.

### Longest common atom strings (LCAS)

We defined the longest common atom string (LCAS) to use as a seed to start multiple alignment. Atom strings were described as the paths consisting of the KEGG atoms and the self-DMAID, of which the longest common (or shared) in the given set of molecules were taken as the LCAS. The procedure of finding LCAS is as follows. First, every atom was regarded as an atom string with the length = 1, and was put into a queue. These atom strings were distinguished by the two properties: the KEGG atom labels and their self-DMAIDs. If the atom strings appeared not in all molecules, then they were discarded. Each of the remaining atom strings was picked out of the queue, and the neighboring atoms of the terminal atom of the string were added to generate all possible atom strings that were one-atom longer. All these atom strings were stored into a new queue, and then the strings that appeared not in all molecules were discarded. Each of the remaining atom strings was picked out of the queue, the neighboring atoms of the terminal atom were added to generate all possible atom strings that were one-atom longer, and was stored in a new queue. This process was iterated until the longer strings shared by all molecules cannot be found any more.

If every one of the molecules had only one LCAS, then the set of LCAS was represented as the “seed” alignment to obtain the core substructure. If there were more than one possible LCAS per molecule (as shown in Figure 2d), the best combination of LCAS was selected as the representative LCAS in the following way. The similarity scores among LCAS were calculated according to the DECAF scores (explained in the next section), and the LCAS that show the best score with other molecules was selected as the representative LCAS of the query molecules. Since there may be too many combinations of LCAS, the suboptimal combination was selected by means of genetic algorithm. Figure 2d shows the LCAS obtained from the two molecules C00482 and C05855. There were two strings per molecule, which were not distinguishable because of the symmetry of the molecules. In this case, any one of the strings could be selected as the representative LCAS.

### Distance-embedded common atom fingerprint (DECAF)

The distance-embedded common atom fingerprint (DECAF) was defined for each vertex in the molecular graphs, as described in Figure 2e. This fingerprint has two attributes: distances d (shortest path length) and the atom strings k. First, the shortest paths among atoms were calculated using a simple width-first search (These were calculated only a single time, and were also used in calculating DMAID). Then the paths were represented as the atom string in backwards, where only the destination atom was described as the atom class. Taking the two molecules in Figure 2 as example, DEKAF for the atom 1 of the molecule C00482 consists of 11 atom strings with their occurrence numbers. Distance = 0 means the atom 1 itself, for which the atom class is “C8”. Distance = 1 refers to the 2-atom-length strings starting from the atom 1, which terminate at the atoms 2, 6 and 12. Distance = 2 refers to the 3-atom-length strings from the atom 1, terminating at 3, 5 and 13. This procedure continued until the width-first search ends, and iterated for all atoms in the molecule.

We defined the following DECAF similarity score, the similarity between atoms, based on the number of common atom strings at each length:

where ni,k,d and nj,k,d are the numbers of atom strings k in the distance d (shortest path length) from the atoms i and j, respectively. Two example DECAFs are shown in Figure 2e, where the common attributes are highlighted in gray. The DECAF scores between the atom 1 of C00482 and the atom 1 of C05855 becomes 1/1 + (1+2)/2 + (1+2)/3 + 1/4 + 1/5 = 3.95. This score was used as a basis of selecting the representative LCAS. This atom-to-atom similarity scores do not have to be calculated between all possible atom pairs in all molecules; they had to be calculated only once when needed at the first time, and then stored in a hash table for the quick use next time.

### Extending the core chemical alignments

All atoms in LCAS were represented in the form of the alignment describing the part of the core substructure. The neighboring atoms of LCAS were picked out, and were grouped by the two properties: the atom species and the anchored-DMAID against LCAS. The atom was discarded if there were any molecule not having the same atom in terms of the two properties. If the atom was unique in a molecule in terms of the two properties, and if the atoms having the same properties uniquely throughout all the query molecules, then the atoms were regarded to be in the core substructure and were added to the alignment. If there were some atoms in a molecule that could not be distinct in terms of the two properties, then the numbers of bonds within ring structures were taken into account. If the atoms were not still distinguished, then the atom classes were additionally considered. If the atoms were not still distinct, then the full KEGG atom types were taken into consideration. In the cases where some atoms could not still be distinguished, occurring when the query molecules contained symmetry, the atoms were distinguished by the order of the numbering. In this way, after the atoms became unique in a molecule in terms of the two properties, and were found in all molecules, then the atoms were added to the core substructure. Among the newly added alignment atoms, the neighboring atoms that were not yet involved in the core alignment were picked out, and the same process were iterated until there found no more atoms.

As the result of the extension of LCAS, the chemical alignment (atom-atom mapping) of the core substructure was obtained. If the query contains only two molecules, the calculation finished here, and output the atom-atom mapping as shown in Figure 2f. If there were more molecules, the search for the branch substructures began as described in the following section.

### Common atom strings for the branch substructures

The branch substructures were obtained with the similar strategy as that for the core substructure. The first step was to obtain many short common atom strings (SCAS) consisting of the atoms that were not involved in the core substructure, instead of the LCAS for finding the core substructure. These strings were distinguished by the atom species and the anchored-DMAID against the core substructure. Different from LCAS, SCAS did not have to involve all the molecules given as a query. Note that SCAS may involve the atom-atom mappings taken from more than two molecules. The next step was to extend the SCAS to obtain the common branch substructure, by the means similar to the extension stage of the LCAS. The difference was that the branch alignments only contains less numbers of molecules than given in the query. The length of the SCAS was not pre-determined, but started from 1 and extended as much as it could go in a greedy fashion.

### Removal of the conflicting atom-atom mapping

After many SCAS were generated independently, there sometimes occurred the cases where different SCAS possessed the same atom. Therefore it was necessary to remove these conflicts. The process of removing the conflicts was as follows: (1) Atom-atom mappings (the columns in the alignment) were ordered randomly. (2) An atom-atom mapping was picked out from the alignment, and was put in a new array. (3) Next atom-atom mapping was picked out, and if it conflicted with the mappings that are already in the array, it was discarded. Otherwise it was added to the array. (4) The step 3 was iterated until the end. (5) The score was defined as the total number of the atoms in the array. (6) The steps 1-5 were repeated 20 times and choose the array with the highest score.

## Results

### Output comparison

**Figure 3:**
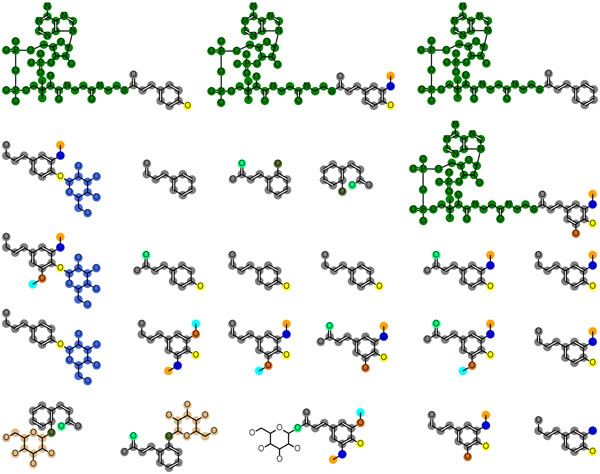
**Example output of MUCHA alignment.** Different colors represent different substructures or building blocks. The colors were given consistently with Figure 5, but not with Figure 4.

**Figure 4:**
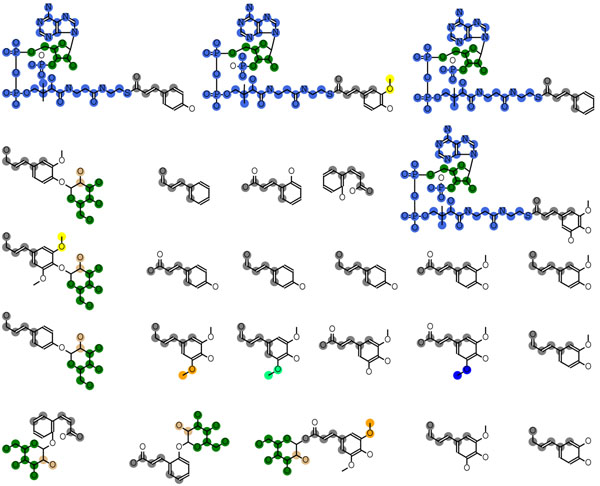
Example output of MULCOMP alignment

MUCHA output the text file that resembled a sequence alignment (Figure 2f), which could be visualized on the chemical structures as shown in Figure 3. The metabolites in Figure 3 are monolignol and related compounds, which are the key metabolite group for phenylpropanoids biosynthesis including lignins, lignans, flavonoids and coumarins. Different colors in Figure 3 indicate the different substructures. It was clearly shown that MUCHA method appropriately divided the query molecules into the substructures, whereas the naïve MULCOMP failed (Figure 4). The naïve method did not take into account the relative distances or positions between the substructures, such as the one in the core substructure (colored in gray) and the one in the sugar residues (colored in green), which resulted in the misalignment of the sugar residue in the different positions. The symmetry around the benzene ring (and the phosphate in the CoA residues as well) caused another problem in the naïve method. Many molecules have more than one atoms that are not distinguishable because of the symmetry. When conducting pairwise chemical alignments, these atoms do not have to be distinct. Since the naïve MULCOMP method iterated the independent pairwise alignments, these atoms were mapped without considering the consistency with the other pairwise alignments. As the result, the naïve method failed to obtain the core structure. The proposed MUCHA method did not conduct the alignment in a pairwise fashion but focusing on finding the core substructure at first, resulting in the better alignment that were consistent throughout all the molecules given as the query. Another advantage of the proposed method was that it discriminated the branch substructures that had the same chemical structure but are attached in different positions, which could not be achieved by simply applying the frequent subgraph mining technique.

### Mapping the branch substructures to pathway

**Figure 5:**
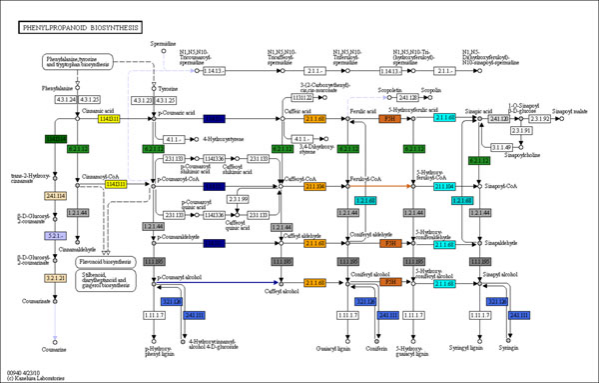
**Mapping substructures to pathway.** The colors were given consistently with Figure 3, but not with Figure 4.

We also found that the alignment obtained by MUCHA correlated well to each reaction step in the metabolic pathway. Figure 5 shows an example pathway colored in accordance with the colors in the branch substructures in Figure 3. This pathway contains some transferase reactions, where the transferred groups (building blocks) were consistent with the branch substructures. It was clearly shown that the branch substructures in the same position were transferred at the similar position in the grid-shaped pathway. These results suggest that the MUCHA alignment gives valuable information to reconstruct metabolic pathways when applied to orphan metabolites.

### Performance evaluation and comparison

**Figure 6:**
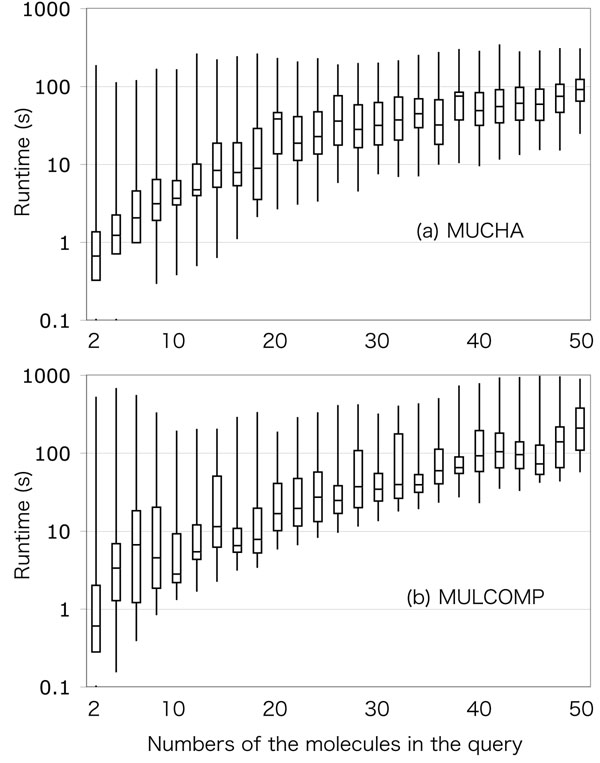
**Runtime of MUCHA and MULCOMP**. The boxplots represent the minimum, lower quartile, median, upper quartile, and maximum of the runtime of (a) MUCHA and (b) MULCOMP calculated for even numbers of metabolites.

**Figure 7:**
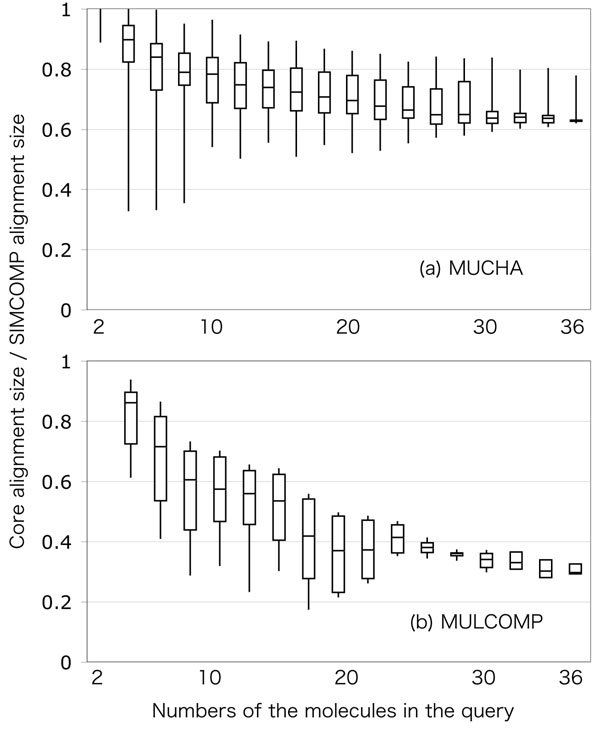
**SIMCOMP alignment retrieval rate of MUCHA and MULCOMP.** The boxplots represent the minimum, lower quartile, median, upper quartile, and maximum of the core alignment size by (a) MUCHA and (b) MULCOMP calculated for monolignols. The core alignment size is normalized by dividing by the average alignment size of the SIMCOMP pairwise alignment for the same metabolite group.

Performance of the two methods has been evaluated in terms of speed (Figures 6 representing the result for all metabolite groups) and quality (Figures 7 for a “monolignol” group of metabolites). The comparison experiment was performed as following. A number of secondary metabolites were retrieved from the KEGG COMPOUND database, and were classified into 34 groups by the KEGG BRITE hierarchical classification. From the obtained metabolite groups, the molecules that have less than 0.5 SIMCOMP similarity score against all other molecules were removed. The resulted molecules contained 43.8 vertices in the KCF representation, corresponding to about 74.0 atoms including hydrogen in average. Multiple chemical alignments by MUCHA and MULCOMP were calculated for different numbers of molecules randomly selected from the metabolite groups.

**Figure 8:**
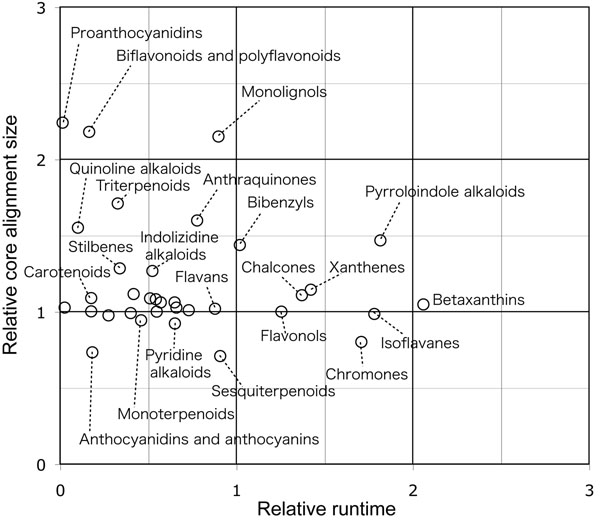
**Relative performance of MUCHA compared with MULCOMP.** The horizontal and vertical axes represent the average runtime and the average core alignment size by MUCHA divided by that of MULCOMP, respectively. If the relative runtime is less than 1, MUCHA ran faster than MULCOMP. If the relative core alignment size is more than 1, MUCHA resulted in larger core substructure than MULCOMP.

In Figures 6, the horizontal axis represent the numbers of molecules in a query, and the vertical axis represent the computational amount in seconds. In Figures 7, the horizontal axes are the same as in the previous figures, but the vertical axes are the relative alignment sizes, which means the size of the core substructure in the multiple alignment divided by the average alignment size of the independent SIMCOMP alignments. The comparison was also represented in Figure 8, where each dot represents the average performances for each metabolite group. In this figure, the relative computational time in the horizontal axis means the average computational time by MUCHA divided by that of MULCOMP. Similarly, the relative alignment size in the vertical axis means the average core alignment size calculated by MUCHA divided by that of MULCOMP.

As shown in Figures 6, runtimes of both methods increased according to the numbers of molecules to be aligned. MULCOMP ran stable in terms of the minimum runtime, although some calculations exceptionally took much time. On the other hand, the runtime of MUCHA was not stable: i.e., it varied depending on the metabolite group (as shown in Figure 8). The runtime of MUCHA was generally less than that of MULCOMP. This runtime may be comparable or a little better than the work by Fober et al., resulted in about 1,000 seconds to calculate multiple chemical alignment of 32 compounds consisting of 48-100 atoms in average [15] (although we cannot determine which is better because their purpose of the multiple chemical alignment was different from ours). Additionally, Figures 7 clearly demonstrate the difference in finding the core substructure. The sizes of the obtained core substructures were relatively stable in MUCHA, whereas they were not in MULCOMP. There were some metabolite groups that showed less performance than MULCOMP in terms of computational time or the core alignment size. However, as a whole, our results indicated that MUCHA alignments showed favorable in finding metabolic building blocks in many metabolite groups (Figure 8).

## Discussion

The SIMCOMP pairwise chemical alignment method was optimized to find a small number of as large as possible substructures, rather than finding a large number of relatively small common substructures. Therefore, simply combining the pairwise chemical alignment results was not efficient for multiple alignment in both terms of computational amount and quality. In this study, the MUCHA method was designed for the multiple chemical alignment, and showed relatively high performance compared with the naïve extension of the pairwise alignments. Apparent performance trade-off exists in some stages, such as the definition of the DECAF score similarity, the genetic algorithm when choosing the representative LCAS, and removing conflicting atom-atom mappings at the end of the alignment. Optimization of these remains to be further argued, although the algorithms and parameters in this work produced reasonable results upon manual inspection.

The performance of the multiple chemical alignment depended upon the choice of the query molecules. As shown in Figure 8, it has been shown that MUCHA did not show better performance in some molecule groups such as sesquiterpenoids, whose chemical structures are highly diverse. If the structures of the given molecules were too diverse, then there would be no common substructures, or the program ended up finding inadequate substructures. In fact, this has been also a problem occurring in the multiple *sequence* alignment methods. As it is important to exclude the sequences that are not evolutionary close when conducting multiple sequence alignments, it is important to exclude the molecules that are not structurally close when conducting multiple chemical alignments. Multiple sequence alignment has a long history, and many researchers have dealt with this problem. This will be one of the problems to solve to better use the multiple chemical alignment.

Although the method to collect the appropriate set of metabolites still remains to be solved, MUCHA had the strength in finding the building blocks for the metabolites that are appropriately collected in advance. The naïve multiple alignment method had to compare the global chemical structures N (N – 1) / 2 times (where N refers to the number of the query molecules) based on the time-consuming clique-finding technique, whereas the MUCHA only needed to compare the local chemical structures by the quick check of the differences among the DECAF vectors, which had been pre-calculated based on the quick width-first search. Local features of graphs can be described by paths or walks (random walks) [30], however, calculation to obtain all random walks consume large computational amount. In this paper, we demonstrated the usefulness of the DECAF similarity scores as the alternatives that can be quickly calculated. One can also easily imagine that the pairwise alignment-based approach would meet the combinatorial explosion with the increasing number of the query molecules, and would also meet the difficulty in keeping the consistency among the pairwise alignments. MUCHA method effectively dealt with these problems by applying the LCAS strategy: the more molecules are given as the query, the less the number of the common atom strings become.

It should be noted that we have to be careful when interpreting the alignment result of orphan metabolites for the metabolic pathway prediction, since the obtained substructures may vary depending on the numbers of the molecules in a query. For example, Figures 1 and 3 show the alignments obtained from the different numbers of the molecules in the same group of the secondary metabolites. Whilst some substructures are the same in these two figures, the substructures of O-methyl groups were divided differently. Looking only at Figure 1, one might think that the O-methyl groups are possibly added to the benzene rings in a single reaction, however, such an enzyme reaction was not found in the KEGG database nor the IUBMB's Enzyme List. It is more natural that a hydroxy group is induced in the benzene ring first, followed by the methylation. This knowledge is consistent with the result shown in Figure 3, where the oxygen atoms and the methyl carbons belong to the separate substructures. This knowledge regarding to the reaction patterns have not been implemented in MUCHA but has implemented in many methods for the metabolic pathway prediction such as E-zyme [31,32], UMPPS [33], GREP [4] and PathPred [34]. Thus we propose that MUCHA and the other methods could complement each other to improve the pathway prediction.

## Conclusion

We provided the method to divide a given set of many molecules into some substructures with descriminating the positions, which has the potential to help automatically classify the metabolites based on the possible origin pathways. The continuous improvement of this method could lead to the reduction of the computational amount when predicting the metabolic pathway, filling the gaps between the metabolomics studies and other omics including genomics, transcriptomics and proteomics.

## Competing interests

None declared.

## Authors' contributions

MKO conceived of the study, designed the algorithm, tested the performance and drafted the manuscript. TT helped the manual inspection of the output results and designed the algorithm. MKA participated in the design of the study. SG helped to design the algorithm and application, and to draft the manuscript. All authors read and approved the final manuscript.
